# Providing longer post-fledging periods increases offspring survival at the expense of future fecundity

**DOI:** 10.1371/journal.pone.0203152

**Published:** 2018-09-10

**Authors:** David López-Idiáquez, Pablo Vergara, Juan Antonio Fargallo, Jesús Martínez-Padilla

**Affiliations:** 1 Department of Evolutionary Ecology, Museo Nacional de Ciencias Naturales, Madrid, Spain; 2 Department of Ecology, Universidad Complutense de Madrid, Spain; 3 Research Unit of Biodiversity (OU, CSIC, PA), Oviedo University, Mieres, Spain; University of Tulsa, UNITED STATES

## Abstract

The cost of reproduction is a key concept in life-history trade-offs. However, our understanding of the reproductive costs is biased towards measures of reproductive effort obtained before offspring independence. During the post-fledging dependence period (PFDP), it is well known that parents feed and protect their offspring. However, the effort devoted to this reproductive activity has never been considered in the context of of the costs of reproduction. Moreover, the potential fitness benefits and costs for offspring and parents, respectively, of the duration of the PFDP are largely unknown. We estimated the duration of the PFDP over 5 years using wild common kestrels (*Falco tinnunculus*) and studied its association with survival probability and future parental reproductive performance. Our results show that longer PFDPs increase the survival probability of fledglings, probably due to the benefits obtained from parental care. In addition, we found that providing longer PFDPs was associated with reduced clutch sizes but not the number of fledglings in the subsequent breeding season in males. We suggest that increased parental expenditures on offspring during the PFDP may represent a potential cost of reproduction in breeding males.

## Introduction

Life history theory posits that since resources are limited, the different strategies or life-history traits may be bound together through trade-offs, whose balance determines individual fitness [1]. The effect of current on future reproduction is one of the best studied trade-offs across different taxa [2–7]. Life-history trade-offs are mediated by the costs incurred from simultaneously producing and maintaining different traits [3,4,8–10]. Particularly, the cost of reproduction implies that increasing current reproductive effort will reduce future reproduction or survival [1]. Given the costs of parental care [11], adult breeders may be subject to reduced survival prospects or to lower reproduction in future breeding attempts [12,13]. However, our understanding of the influence of parental care and their associated costs is based almost entirely on observations and experiments made on parental expenditure during the pre-nesting and nesting stages [3,4,10,14]. This is particularly relevant in birds, a taxonomic group that has been widely used to build the life-history theory. Birds exhibit parental care after the nesting stage and yet, parental expenditure during this phase has rarely been considered in research on the costs of reproduction and life history. In birds, the period during which the fledglings become independent is a crucial stage that may greatly determine offspring fitness [15]. It demands a considerable amount of parental resources [13] and shows variable duration among species and individuals [15,16]. However, we know rather little about the potential cost for parents of a prolonged stage of parental care during the post-fledging dependence period (PFDP).

During the PFDP, fledglings obtain direct benefits from their parents in terms of food [16], nutritional status improvement [17,18], competitive capacity [19] or acquiring foraging and flying skills [20–25], potentially improving survival prospects [17,26]. While very few and recent examples confirm that higher parental expenditure results in a longer PFDP [16,27], the potential reproductive cost of extended parental care paid later in life has been scarcely explored [13].

Theoretically, a parent-offspring conflict emerges because each offspring within a family demands greater parental investment since it is more related to itself than to its siblings, while genetic similarity between parents and all offspring does not differ [28,29]. This differential within-brood allocation during the PFDP has been associated with phenotypic traits, such as melanin-pigmented plumage traits, sex and age [16,27]. These differences can arise due to differential parental investment according to the expected fitness return of each fledgling [30]. For example, parents can invest more in the dispersing sex when there is limited resource availability in order to avoid future competition [31]. Thus, in light of the parent-offspring conflict, it would be expected that an increased parental expenditure during the PFDP will increase survival prospects of offspring, but also impose a reproductive cost to breeders paid through reduced survival and/or future reproduction.

In this study, we explored the fitness consequences of the length of the PFDP of 315 fledglings of Common Kestrels (*Falco tinnunculus*) over 5 years. We also explore the potential fitness costs of the length of the PFDP on parents in the subsequent breeding season. The duration of the PFDP is variable in kestrels [10,19] and we base our predictions on the idea that PFDP is costly for breeding parents. We therefore predict that 1) parents raising offspring with longer PFDPs will pay a fitness cost, either in reproduction or survival and 2) longer PFDPs will increase survival rates of fledglings.

## Methods

### Ethics statement

All licences legally required to study, capture and handle kestrels were provided by the Regional Government of Castilla y León (Ref:201715700010993), where our population is located. In addition, we held all the necessary licenses for capturing kestrels approved by the bioethical committee of our research institute, Museo Nacional de Ciencias Naturales (CSIC). Blood samples were taken following standard procedures as previously described in our kestrel population. During capture, handling or bleeding, we never observed any adverse effect either in adults or in offspring. Finally, fieldwork was conducted on private land under the permission of the owners, the Finat Family.

### Study species

The Common Kestrel is a medium-sized diurnal raptor that exhibits a marked sexual dimorphism in body size and plumage colouration [32–34]. At the beginning of the breeding season, males perform courtship feedings increasing their hunting effort by two-fold or more [32,35]. The time that these courtship feedings begin has a strong influence on the time that females start laying eggs, egg quality, and clutch size [32,36–38]. Food provisioning during the breeding season is done nearly exclusively by males [19,32,39,40], while females mainly incubate the eggs [32]. Male food provisioning is maintained throughout nearly the entire breeding season until the second half of the nestling stage when females begin hunting, supporting males in feeding the brood [32]. Fledgling plumage is similar to females, displaying a variable proportion of grey colouration on the rump, a trait that has been described as an index of quality and that is associated with the duration of the PFDP [19,41].

### General procedures

The study was conducted from 2005 to 2017 in the Campo Azálvaro region, a flat and treeless grassland located in central Spain mainly devoted to cattle grazing (40°40’N, 4°20’W). During the breeding season, nests were monitored to detect laying date (day the first egg was laid) and to record clutch size (mean = 4.98, range = 3–7, n = 86) and number of fledglings (mean = 4.13, range = 1–6, n = 86). Adult breeders were captured using nest traps when nestlings were 10–13 days old. When nestlings were 26 days old, they were weighed (to the nearest g) and their blood was sampled for molecular sexing. In addition, we also measured the proportion of grey colouration on nestling rumps (0% corresponding to a completely brown rump and 100% to a fully grey rump [42]). Nestlings were marked with a combination of colour rings during 2005–2007 and 2012 and with PVC rings with a unique alphanumeric code during the year 2013 to identify fledglings from long distances [16]. We carried out a food-supplementation experiment in 2006 [16], and only considered the un-manipulated individuals in this study.

### PFDP duration, offspring and adult survival

We consider the length of the PFDP as the number of days that fledglings remain in the surroundings of the nest, with 31.3 days old being the average age at which the chicks begin fledging [19]. The duration of the PFDP was estimated for 315 fledglings from 86 nests during the months of June, July and August for 5 years (21 nests in 2005, 13 in 2006, 21 in 2007, 19 in 2012 and 12 in 2012) by identifying all fledglings present around the nest every two days. In our study area, fledglings are easily detectable from long distances. Breeding nest-boxes are located alongside fences and fledglings are usually seen perching on them rather than on the ground. Fledgling identification was made from a car, using a telescope (20x60) between 7:30 and 19:30, at a distance sufficient to identify the coloured rings or to read the PVC rings without interfering with fledgling behaviour (see [19] for further information). We considered “day of independence” as the last day an individual was observed and the duration of the PFDP as the number of days since chicks were 32 days old (age at which all nestling have fledged [19]) until the day of independence. Nest monitoring was prolonged for 7 days from the last detection of a given offspring to increase the accuracy of the PFDP duration estimation. This estimation of the duration of the PFDP can be considered misleading since mortality before independence can alter the estimation of the length of the PFDP. However, a recent review reported that weekly survival rates for predator species during the first month are around 90% [43]. In addition, during the long-term monitoring of our population, we detected an extremely low rate of predation during the PFDP (9 out of 1737 fledglings ringed from 1998 to 2016). Thus, we consider that our estimation of the duration of the PFDP is highly reliable, as previously shown [16,19].

We used recruitment as a proxy of post-fledging survival [10,44–46]. In our population, fledgling recruitment takes place during the first four years of life (first year 44%, second year 48%, third year 6% and fourth year 2%; n = 130, period between 2004 and 2017). However, 92% of the recruits occurred during the 2 years following birth. Recruitment has never been observed after four years of age. For this reason, we monitored the four years following the year of birth to determine fledgling survival. In addition, kestrels in our population do not skip breeding. Only seven out of 153 individuals (5%) that nested two years or more in our population were missing in any intervening year, although the absence of these individuals was probably due to the impossibility of capturing them rather than a skipped breeding, as some nests can fail before capture. Adult survival was then estimated by their presence-absence as breeders during the two subsequent years.

Both fledgling and adult survival were estimated by banding individuals with PVC and metal ring codes and recapturing them in the subsequent years as breeders (using nest-traps when nestlings are 10–13 days old), using nest camera recorders or reading ring PVC codes with the aid of a telescope when it was not possible with either of the two first methods (see [47]).

### Statistical procedures

All models were conducted in R statistical software using the *lme4* and *car* packages [48,49]. We performed three sets of models. The first set of models aimed to explore the association between the duration of the PFDP and survival in offspring, using Generalised Linear Mixed Models (GLMM) with binomial distribution of errors. Survival (1 or 0) was the dependent variable and PFDP, chick sex, rump colouration, laying date and weight were included as explanatory terms. Interactions between PFDP and sex and PFDP and rump colouration were also included in the models. In addition, we performed a model to explore the association between clutch size and the number of fledglings and the duration of the PFDP. We performed a Linear Mixed Model (LMM) with normal distribution of errors in which PFDP was included as a dependent variable, and laying date, clutch size, fledgling weight and sex, and the number of fledglings as explanatory terms. In both models, since food conditions vary inter-annually [50] and due to the low number of levels of this variable (5 levels corresponding to each study year), year was included in the model as a fixed factor. Female identity was included as a random factor. We used female instead of nest because female quality may mediate the duration of the PFDP [16] and because the same female produced offspring in different years.

The second set of models was carried out to explore the association between the duration of PFDP experienced by breeding adults in a given year (*t*) and their reproductive output the following year (*t+1*). We used LMMs with normal distribution of errors considering clutch size (CS_*t+1*_), and the number of fledglings (NF_*t+1*_) as dependent variables in different models. Since breeding adults in a given year (*t*) may have raised offspring with different PFDP durations, we used the mean duration of the PFDP of the brood for each adult each year (PFDPmean_*t*_) as an explanatory variable. To control for the potential effect of current reproductive effort, we included clutch size (CS_*t*_) and the number of fledglings (NF_*t*_) in year (*t*) as explanatory variables when CS_*t+1*_ or NF_*t+1*_ were the dependent variables, respectively. Laying date (LD_*t+1*_) was also included in all models to control for the differences in reproductive performance across the breeding season. Previous studies in this species have shown that clutch size is positively associated with age in males [51]. To control for the potential effects of this variable we included minimum age as an explanatory variable. We included this term of minimum age because we do not reliably know the real age of all the individuals and including the latter would cause a reduction in the number of observations included in the models. Finally, year was included as a fixed factor to control for the inter-annual differences in food abundance (see above). Individual identity was included as a random factor.

In the third set of models, we explored the association between the duration of the PFDP and adult survival to the following breeding season for males and females separately. We included adult survival as a dependent variable and PFDPmean_*t*_ as an explanatory term, using GLMMs with a binomial distribution of errors. We also included CS at year *t* as a covariate to control for the potential effects of past parental expenditure on adult survival. Year was included as a covariate to control for the potential effects of environmental heterogeneity [50] and individual identity as a random factor. In addition, males and females were analysed separately as they play different roles during breeding and thus they may experience different reproduction costs.

In all models, we calculated the Variance Inflation Factors (VIFs) among our explanatory variables and found no evidence of collinearity (all<1.43—see S1–S8 Tables).

## Results

**Fig 1:**
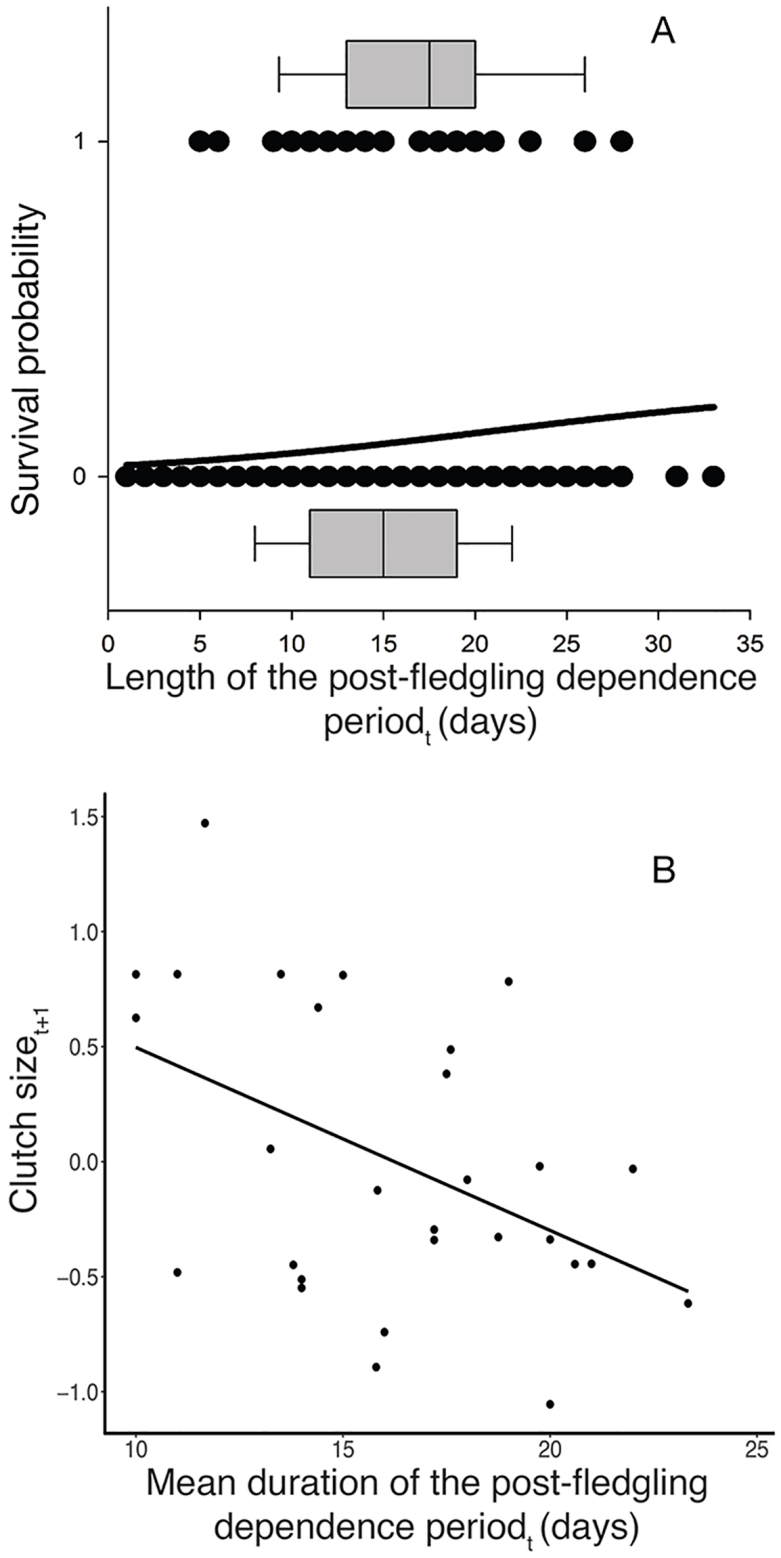
(A) Relationship between the probability of post-fledging survival and the duration of the post-fledging dependence period measured in days. Dots represent each individual fledgling and the box plot represents the mean length of the post-fledging dependence period in days for survivors (survival probability = 1) and non-survivors (survival probability = 0). In (B), we show the relationship between clutch size and mean duration of the post-fledging dependence period. Values of clutch size represent the residuals obtained from the model (see Methods for further details). In both figures, sub-indices denote current (_*t*_) or following year (_*t+1*_).

We found that the mean duration of the PFDP for the kestrel fledglings was 15.25±0.40 days. Regarding offspring, we found that the length of the PFDP was positively associated with the probability of offspring survival (0.067±0.034, F = 1.942, *P* = 0.049, Fig 1). Year was retained as a covariate as two of its levels were significantly associated with the probability of survival (Year_2006_, p = 0.040; Year_2013_, *P* = 0.044). None of the explanatory variables (chick sex: F = 0.021, *P* = 0.647; rump colouration: F = 0.518, p = 0.414; laying date: F = 0.535, *P* = 0.531; weight: F = 0.761, *P* = 0.380) or interactions (PFDP*chick sex: F = 0.869, *P* = 0.351; PFDP*rump colouration: F = 0.395, *P* = 0.535) explained the probability of survival. We also found a significant positive association between PFDP and clutch size (1.478±0.571, F_1,120.910_ = 6.685, p = 0.010) but not for the number of fledglings (F_1,166.220_ = 1.097, p = 0.296). In the model, laying date (-0.235±0.049, F_1,132.828_ = 22.564, p<0.001) and year (F_4,147.16_ = 9.392, p<0.001) were retained as statistically significant covariates. No significant associations were found either for fledgling weight (F_1,271.925_ = 0.039, p = 0.842) or for sex (F_1,276.32_ = 0.086, p = 0.769).

In relation to breeding adults, we found that the mean length of the PFDP and clutch size the following year (*t+1*) were negatively associated (Table 1, Fig 1) in males. No relationships were found either for NF_*t+1*_, or for the reproductive variables explored in females (Table 1). We found no significant relationship between PFDPmean_t_ and adult survival (Table 2). In both models, the results remained unchanged when considering the maximum duration of the PFDP (see S9 and S10 Tables for further information).

**Table 1:** Results of the Linear Mixed Models exploring the association between the mean duration of the post-fledging dependence period (PFDPmean_*t*_) and the following years' (Year_*t+1*_) clutch size (CS_*t+1*_) and number of fledglings (NF_*t+1*_).

	Females (n = 43)					Males (n = 29)				
	CS_t+1_									
Parameter	Estimate	SE	*F*	*P*	*E*.*Seq*	Estimate	SE	*F*	*P*	*E*.*Seq*
Mean PF	-0.014	0.028	F_1,34.38_ = 0.254	0.617	3	**-0.104**	**0.036**	**F**_**1,21**_ **= 8.067**	**0.009**	
LD_t+1_	-0.006	0.017	F_1,32.34_ = 0.142	0.708	1	**-0.078**	**0.017**	**F**_**1,21**_ **= 19.090**	**<0.001**	
Year_t+1_			F_4,26.69_ = 2.346	0.080				**F**_**4,21**_ **= 3.327**	**0.029**	
Minage_t+1_	-0.045	0.080	F_1,36.84_ = 0.312	0.579	4	**0.285**	**0.107**	**F**_**1,21**_ **= 7.123**	**0.014**	
CS_t_	0.071	0.173	F_1,31.44_ = 0.169	0.683	2	0.067	0.254	F_1,20_ = 0.070	0.793	1
	NF_t+1_									
Mean PF	0.009	0.063	F_1,22.29_ = 0.024	0.877	1	-0.047	0.058	F_1,20_ = 0.656	0.427	1
LD_t+1_	-0.012	0.039	F_1,34.90_ = 0.101	0.752	2	**-0.056**	**0.020**	**F**_**1,13.12**_ **= 8.008**	**0.014**	
Minage_t+1_	0.182	0.179	F_1,39.88_ = 1.032	0.315	4	**0.451**	**0.180**	**F**_**1,26.00**_ **= 6.215**	**0.019**	
Year_t+1_			F_1,18.55_ = 0.575	0.684	3			F_4,17.81_ = 1.937	0.148	3
NF_t_	-0.334	0.209	F_1,39.02_ = 2.554	0.118		-0.272	0.207	F_1,20.97_ = 1.726	0.203	2

Minage_x+1_ represents the minimum age for an individual in our population. Statistically significant variables are highlighted in bold.

**Table 2:** Results of the Generalised Linear Mixed Models exploring the association between the mean duration of the post-fledging dependence period (PFDPmean_*t*_) and survival to the following reproductive season (*t+1*).

	Females (n = 86)				Males (n = 77)			
Parameter	Estimate	SE	*F*	*P*	Estimate	SE	*F*	*P*
PFDPmean_t_	0.039	0.061	F_1_ = 0.436	0.516	0.092	0.074	F_1_ = 3.328	0.217
Year_t_			F_4_ = 2.562	0.120			F_4_ = 1.084	0.210
CS_*t*_	-0.057	0.362	F_1_ = 0.026	0.873	**1.310**	**0.528**	**F**_**1**_ **= 8.220**	**0.013**

Year and clutch size (CS_*t*_) are included as covariates in the models. Statistically significant variables are highlighted in bold.

## Discussion

Our results reveal opposite relationships between the PFDP and fitness components of parents and offspring. A longer duration of the PFDP seems to increase survival in fledglings on the one hand and reduce future fecundity in parents on the other.

The positive association between the duration of the PFDP and survival may arise from the benefits that offspring obtain during this period. For example, breeding parents in better body condition are able to invest more time and energy in their offspring resulting in longer PFDPs [16]. This investment is expected to increase the body condition of the offspring [17], a key component for recruitment and survival [52]. Also, during this period fledglings spend much of their time practicing hunting with inanimate objects or real prey (pers. obs., [32]). Thus, after longer PFDPs fledglings are expected to improve flying and hunting skills that could potentially increase their survival probabilities [17]. Fledgling sex did not affect the association between the length of the PFDP and survival rates. These results may be unexpected considering that females are superior competitors for food compared to their male sibs during the nestling period [53] and that better body condition at fledging increases offspring survival [52]. In kestrels, during the nestling period, a lower competitive capacity in males results in a reduction in body condition with respect to female fledglings [10,53]. However, there is a shift in the access to food provided by parents during the PFDP [19]. Specifically, males outcompete females in their access to food due to their enhanced flying skills [19]. This result may support the idea that there is a sex-dependent improvement in body condition in male fledglings during the PFDP, compensating for the lower sibling competition abilities of males during the nestling period [53].

It can be argued that our estimates of survival are biased due to the potential confounding effects of dispersing individuals, as it has been described that fledglings can disperse more than 100 km [54], although in adults, 84% of individuals disperse at distances less than 10 km and 94% less than 25 km [46]. Our population is located in an isolated nest-box population area [55] and it has been reported that kestrels positively select nest-boxes for breeding over other nest sites when provided [32,56,57]. Considering that every year during the study period a proportion of nest-boxes are left unoccupied (varying from 31% to 77% in the study period), we believe the dispersal outside our study area must be very low. The distance between the two farthest nest-boxes in our study area is 5 km [48]. Thus, the study of the dispersal between nest-boxes, as conducted in other studies [47, 54], would not be very informative in our case. We are confident in the reliability of our survival estimation, as we did not find any individuals breeding in the surroundings of our study area or any dispersing chicks breeding in any of the nearby nest-box populations (Villalar de los Comuneros– 112 km from our study area; San Martín de Valderaduey– 155 km from our study area) that we also study [58,59]. Nevertheless, for our purposes, natal dispersal is considered a cost in terms of fitness reduction, as it increases mortality [60,61].

Our results also show that males that raised offspring for longer PFDPs, paired up with females that laid smaller clutches the following year. The cost of reproduction implies that increasing current reproductive effort will reduce future reproduction or survival [1]. Our results support this idea, as the potential cost of caring for offspring during the PFDP may be paid in terms of smaller clutches but not in the number of fledglings or adult survival in the subsequent breeding season. This specific life-history trade-off between current and future reproduction has been previously demonstrated in birds [3,4,6], and explained by an increased energy investment during reproduction. We found that a decrease in clutch size with an increase in the duration of PFDP occurred only in males, probably because of the higher resource expenditure of males compared to females in their offspring during the post-fledging period, likely in feeding and parental care [19,40]. In addition, it has been experimentally shown that only breeding males increase their hunting effort as a response to enlarged broods [10]. Thus, the reduced clutch sizes the following breeding season can be explained by the cost that males paid when raising offspring for a longer PFDP the previous year, the effect being only observed during the initial stages of reproduction, when the females are laying eggs. However, our results do not show fitness differences associated with providing longer or shorter PFDPs, as we did not find a significant association between PFDP duration and the number of fledglings. To explain this apparent contradiction, we speculate that males providing longer PFDPs are of higher quality, with these males being more efficient in chick-rearing, which allows them to recover from a potential fitness reduction as a result of the decrease in the number of eggs laid. This agrees with the “hypothesis of clutch size errors” proposed by J.M. Aparicio (1993) [62] This hypothesis posits that the relationship between fitness and parental quality is more dependent on the probability of misadjusting the clutch size with respect to the number of fledglings that the parents can adequately rear, than producing larger or smaller clutches *per se*. Although speculative, this idea is plausible and should be investigated by manipulating the duration of the PFDP.

Finally, our results show that providing longer PFDPs did not have any significant effects on survival probabilities of the breeding adults. Interestingly, however, those analyses also revealed a significant and positive association between clutch size and the probability of surviving to the following breeding season in males. This result suggests that clutch size, rather than the number of fledglings, is a better proxy for male investment in the common kestrel [63]. This association is supported by our results that show that clutch size is a better predictor of the duration of the PFDP than the number of fledglings, in agreement with previous studies stating that clutch size is a good predictor of parental investment in offspring [63] and of male quality [64].

Taken together, our results suggest two opposing selective forces within a parent-offspring conflict context. On the one hand, offspring will be positively selected to extend the duration of the PFDP, as they increase their own probability of survival. On the other, parental effort devoted to increasing the time spent caring for offspring during the PFDP may lead to costs in terms of future productivity by reducing the number of eggs laid the following year. Future studies are needed to understand why the clutch size reduction experienced in males providing longer PFDPs is not reflected in a decrease in the number of fledglings.

## Supporting information

S1 Table Variance Inflation Factors (VIF) for the dependent variables used in the models exploring the association between the mean duration of the post-fledgling dependence period (PFDPmean) and following years (*t+1*) reproductive output in males. (DOCX)

S2 Table Correlation coefficients between each pair of variables used as explanatory in the models exploring the association between the mean duration of the post-fledgling dependence period (PFDPmean) and following years (*t+1*) reproductive output in males. (DOCX)

S3 Table Variance Inflation Factors (VIF) for the dependent variables used in the models exploring the association between the mean duration of the post-fledgling dependence period (PFDPmean) and followings year (*t+1*) reproductive output in females. (DOCX)

S4 Table Correlation coefficients between each pair of variables used as explanatory in the models exploring the association between the mean duration of the post-fledgling dependence period (PFDPmean) and followings year (*t+1*) reproductive output in females. (DOCX)

S5 Table Variance Inflation Factors (VIF) for the dependent variables used in the models exploring the association between the post-fledgling dependence period (PFDP) length on fledgling recruitment. (DOCX)

S6 Table Correlation coefficients between each pair of variables used as explanatory in the models exploring the association between the post-fledgling dependence period (PFDP) length and the reproductive output of the parents. (DOCX)

S7 Table Variance Inflation Factors (VIF) for the dependent variables used in the models exploring the association between the post-fledgling dependence period (PFDP) length and the reproductive output of the parents. (DOCX)

S8 Table Correlation coefficients between each pair of variables used as explanatory in the models exploring the influence of the post-fledgling dependence period (PFDP) length on fledgling recruitment. (DOCX)

S9 Table Results of the Linear Mixed Models exploring the association between the maximum duration of the post-fledging dependence period (PFDPmax_*t*_) and the following years' clutch size (CS_*t+1*_) and number of fledglings (NF_*t+1*_). (DOCX)

S10 Table Results of the Generalised Linear Mixed Models exploring the association between the mean duration of the post-fledging dependence period (PFDPmean_*t*_) and survival to the following reproductive season (*t+1*). (DOCX)
