# Supplementary material for: Providing longer post-fledging periods increases offspring survival at the expense of future fecundity
Source: PLoS One. 2018 Sep 10;13(9):e0203152. doi: 10.1371/journal.pone.0203152 (PMC6130873; doi:10.1371/journal.pone.0203152)
Supplement: S1 Table — (DOCX) [file pone.0203152.s001.docx]

S1 Table

|  | Models | |
| --- | --- | --- |
| Variables | Clutch Size_t+1_ | N. of Fledglings_t+1_ |
| PFDPmean_t_ | 1.082 | 1.001 |
| Laying date_t+1_ | 1.017 | 1.095 |
| Clutch size_t_ | 1.095 |  |
| N. of fledglings_t_ |  | 1.094 |

Table A. Variance Inflation Factors (VIF) for the dependent variables used in the models exploring the association between the mean duration of the post-fledgling dependence period (PFDPmean) and following years (*t+1*) reproductive output in males.
