# Supplementary material for: Providing longer post-fledging periods increases offspring survival at the expense of future fecundity
Source: PLoS One. 2018 Sep 10;13(9):e0203152. doi: 10.1371/journal.pone.0203152 (PMC6130873; doi:10.1371/journal.pone.0203152)
Supplement: S2 Table — (DOCX) [file pone.0203152.s002.docx]

S2 Table

|  | PFDPmean_t_ | Laying date_t+1_ | Clutch size_t_ | N. of fledglings_t_ |
| --- | --- | --- | --- | --- |
| PFDPmean_t_ | 1 | 0.035 | 0.268 | -0.006 |
| Laying date_t+1_ |  | 1 | -0.112 | -0.293 |
| Clutch size_t_ |  |  | 1 | 0.377* |
| N. of fledglings_t_ |  |  |  | 1 |

Correlation coefficients between each pair of variables used as explanatory in the models exploring the association between the mean duration of the post-fledgling dependence period (PFDPmean) and following years (*t+1*) reproductive output in males. Asterisks represent the significance of the correlations (***p<0.001; **p<0.01; *p<0.05).
