# Supplementary material for: Providing longer post-fledging periods increases offspring survival at the expense of future fecundity
Source: PLoS One. 2018 Sep 10;13(9):e0203152. doi: 10.1371/journal.pone.0203152 (PMC6130873; doi:10.1371/journal.pone.0203152)
Supplement: S3 Table — (DOCX) [file pone.0203152.s003.docx]

S3 Table

|  | Models | |
| --- | --- | --- |
| Variables | Clutch Size_t+1_ | N. of Fledglings_t+1_ |
| PFDPmean_t_ | 1.141 | 1.173 |
| Laying date_t+1_ | 1.091 | 1.098 |
| Clutch size_t_ | 1.090 |  |
| N. of fledglings_t_ |  | 1.098 |

Variance Inflation Factors (VIF) for the dependent variables used in the models exploring the association between the mean duration of the post-fledgling dependence period (PFDPmean) and followings year (*t+1*) reproductive output in females.
