# Supplementary material for: Providing longer post-fledging periods increases offspring survival at the expense of future fecundity
Source: PLoS One. 2018 Sep 10;13(9):e0203152. doi: 10.1371/journal.pone.0203152 (PMC6130873; doi:10.1371/journal.pone.0203152)
Supplement: S5 Table — (DOCX) [file pone.0203152.s005.docx]

S5 Table

| Parameters | VIF values |
| --- | --- |
| PFDP | 1.098 |
| Laying date | 1.163 |
| Weight | 1.141 |
| Rump colouration | 1.094 |

Variance Inflation Factors (VIF) for the dependent variables used in the models exploring the association between the post-fledgling dependence period (PFDP) length on fledgling recruitment.
