# Supplementary material for: Providing longer post-fledging periods increases offspring survival at the expense of future fecundity
Source: PLoS One. 2018 Sep 10;13(9):e0203152. doi: 10.1371/journal.pone.0203152 (PMC6130873; doi:10.1371/journal.pone.0203152)
Supplement: S6 Table — (DOCX) [file pone.0203152.s006.docx]

S6 Table

|  | Laying date | Clutch size | Number of fledglings | Weight |
| --- | --- | --- | --- | --- |
| Laying date | 1 | -0.393*** | -0.390*** | -0.219*** |
| Clutch size |  | 1 | 0.506*** | 0.031 |
| Number of fledglings |  |  | 1 | 0.062 |
| Weight |  |  |  | 1 |

Table F. Correlation coefficients between each pair of variables used as explanatory in the models exploring the association between the post-fledgling dependence period (PFDP) length and the reproductive output of the parents. Asterisks represent the significance of the correlations (***p<0.001; **p<0.01; *p<0.05).
