# Supplementary material for: Providing longer post-fledging periods increases offspring survival at the expense of future fecundity
Source: PLoS One. 2018 Sep 10;13(9):e0203152. doi: 10.1371/journal.pone.0203152 (PMC6130873; doi:10.1371/journal.pone.0203152)
Supplement: S8 Table — (DOCX) [file pone.0203152.s008.docx]

S8 Table

|  | PFDP | Laying date | Weight | Rump Coloration |
| --- | --- | --- | --- | --- |
| PFDP | 1 | -0.295*** | 0.052 | 0.069 |
| Laying date |  | 1 | -0.219*** | -0.068 |
| Weight |  |  | 1 | -0.259*** |
| Rump Colouration |  |  |  | 1 |

Correlation coefficients between each pair of variables used as explanatory in the models exploring the influence of the post-fledgling dependence period (PFDP) length on fledgling recruitment. Asterisks represent the significance of the correlations (***p<0.001; **p<0.01; *p<0.05).
