# Supplementary material for: Providing longer post-fledging periods increases offspring survival at the expense of future fecundity
Source: PLoS One. 2018 Sep 10;13(9):e0203152. doi: 10.1371/journal.pone.0203152 (PMC6130873; doi:10.1371/journal.pone.0203152)
Supplement: S9 Table — (DOCX) [file pone.0203152.s009.docx]

S9 Table

It is possible that the costs associated to parental care can arise not only from an increased work load, but also from the duration of the care, for example if expending more time in the breeding areas makes more difficult obtaining high quality territories during winter. To deal with issue, we also fitted the models exploring the costs of the PFDP using the maximum duration of the PFDP (PFDPmax_t_) in each nest, instead of the mean duration of the PFDP (PFDPmean_t_). The results remain the unchanged, for the models of survival and reproduction, in both males and females.

|  | Females (n=43) | | | | | Males (n=29) | | | | |
| --- | --- | --- | --- | --- | --- | --- | --- | --- | --- | --- |
|  | CS_x+1_ | | | | | CS_x+1_ | | | | |
| Parameter | Estimate | SE | *F* | *P* | *E.Seq* | Estimate | SE | *F* | *P* | *E.Seq* |
| PFDPmax_t_ | -0.012 | 0.022 | F_1,36.97_=0.327 | 0.570 | 4 | **-0.072** | **0.022** | **F_1,3,36_=10.508** | **0.040** |  |
| LD_t+1_ | -0.006 | 0.017 | F_1,32.33_=0.133 | 0.717 | 1 | **-0.073** | **0.0193** | **F_1,20.10_=14.6267** | **0.001** |  |
| Year_t+1_ |  |  | F_4,26.697_=2.346 | 0.080 |  |  |  | **F_4,9.17_=4.083** | **0.036** |  |
| Min. Age_t+1_ | -0.043 | 0.081 | F_1,35.890_=0.594 | 0.594 | 3 | **0.264** | **0.116** | **F_1,19.45_=5.216** | **0.033** |  |
| CS_t_ | 0.070 | 0.173 | F_1,31.97_=0.163 | 0.688 | 2 | 0.090 | 0.282 | F_1,19.24_=0.103 | 0.750 | 1 |
|  | NFx+1 | | | | | NFx+1 | | | | |
| PFDPmax_t_ | 0.003 | 0.052 | F_1,27.35_=0.005 | 0.944 | 1 | -0.033 | 0.040 | F_1,20.0_=0,690 | 0.415 | 1 |
| LD_t+1_ | -0.012 | 0.039 | F_1,34.90_=0.101 | 0.752 | 2 | **-0.056** | **0.020** | **F_1,13.123_=8.008** | **0.014** |  |
| Min. Age_t+1_ | 0.182 | 0.179 | F_1,39.88_=1.032 | 0.315 | 4 | **0.451** | **0.180** | **F_1,26.0_=6.215** | **0.019** |  |
| Year_t+1_ |  |  | F_4,18.855_=0.575 | 0.684 | 3 |  |  | F_4,17.812_=1.937 | 0.148 | 3 |
| NF_x_ | -0.334 | 0.209 | F_1,39.02_=2.554 | 0.188 |  | -0.272 | 0.207 | F_1,20.973_=1.726 | 0.203 | 2 |

Results of the Linear Mixed Models exploring the association between the maximum duration of the post-fledging dependence period (PFDPmax*_t_*) and the following years' clutch size (CS*_t+1_*) and number of fledglings (NF*_t+1_*). Year_t+1_ was introduced as a covariate. Minage_x+1_ represents the minimum age for an individual on our population. Statistically significant variables are highlighted in bold. Values for excluded variables refer to the step before their exclusion (E.Seq).
