# Supplementary material for: Providing longer post-fledging periods increases offspring survival at the expense of future fecundity
Source: PLoS One. 2018 Sep 10;13(9):e0203152. doi: 10.1371/journal.pone.0203152 (PMC6130873; doi:10.1371/journal.pone.0203152)
Supplement: S10 Table — (DOCX) [file pone.0203152.s010.docx]

S10 Table

|  | Females (n=86) | | | | | Males (n=77) | | | | |
| --- | --- | --- | --- | --- | --- | --- | --- | --- | --- | --- |
|  | Survival | | | | | | | | | |
| Parameter | Estimate | SE | *F* | *P* | *E.Seq* | Estimate | SE | *F* | *P* | *E.Seq* |
| PFmax_t_ | 0.011 | 0.046 | F_1_=0.069 | 0.798 | 2 | 0.072 | 0.063 | F_1_=3.798 | 0.256 | 1 |
| Year_t_ |  |  | F_4_=10.249 | 0.120 |  |  |  | F_4_=1.084 | 0.210 | 2 |
| CS_t_ | -0.001 | 0.369 | F_1_=0.000 | 0.998 | 1 | **1.310** | **0.528** | **F_1_=8.220** | **0.013** |  |

Results of the Generalised Linear Mixed Models exploring the association between the mean duration of the post-fledging dependence period (PFDPmean*_t_*) and survival to the following reproductive season (*t+1*). Year and clutch size (CS*_t_*) are included as covariates in the models. Statistically significant variables are highlighted in bold. Values for excluded variables refer to the step before their exclusion (E.Seq).
